# Revolutionizing cancer treatment: The multifaceted role of graphene oxide in modern oncology

**DOI:** 10.1002/btm2.70055

**Published:** 2025-07-30

**Authors:** Yutong Wu, Ting Zhu, Kou Wu, Zean Wang, Sizhe Jiao, Jiaxin Li, Weihong Guo, Xiaoli Feng

**Affiliations:** ^1^ Stomatology Hospital, School of Stomatology Southern Medical University Guangzhou China; ^2^ Department of General Surgery Nanfang Hospital, Southern Medical University Guangzhou China

**Keywords:** biocompatibility, cancer therapy, clinical transformation, drug delivery, graphene oxide, nanomaterials

## Abstract

The rising global incidence and mortality rates of cancer underscore the persistent reliance on chemotherapy as the primary treatment modality. Despite its widespread use, challenges such as chemotherapy resistance and the absence of tumor‐specific targeting have limited its efficacy, thereby necessitating the development of more effective therapeutic strategies in clinical practice. In this context, nanomaterials have opened up new avenues for cancer therapy. Among these, nanoparticles like graphene oxide (GO) exhibit significant potential due to their large specific surface area, high biocompatibility, abundance of oxygen‐containing functional groups, and exceptional biocompatibility. This review systematically summarizes the intrinsic antitumor properties of GO and emphasizes its role in enhancing the delivery and therapeutic efficacy of chemotherapeutic agents, gene drugs, and natural compounds through multiple mechanisms. It further highlights GO's potential in synergistic chemotherapy, targeted therapy, tumor monitoring platforms, and cancer vaccine development, while also discussing the manufacturing challenges that limit clinical translation, aiming to provide theoretical guidance and innovative strategies for its future application in oncology.

**Translational Impact Statement**

Chemotherapy resistance and poor tumor targeting hamper conventional treatments. With its large surface area and functional versatility, graphene oxide (GO) acts as an advanced nanocarrier to enhance the delivery of diverse therapeutics, overcome resistance, improve biocompatibility, and modulate antitumor immunity. This review delineates GO's applications in synergistic chemotherapy, targeted therapy, tumor monitoring, and vaccine development, and analyzes current clinical translation challenges, aiming to advance GO‐based nanomedicines toward oncology practice.

## 1 INTRODUCTION

According to the American Cancer Society, by 2025, there will be an estimated 2,041,910 new cancer cases and 618,120 cancer deaths occurring in the United States,^1^ underscoring that cancer remains a major problem that seriously threatens the lives of people worldwide. Currently, chemotherapy is the cornerstone of cancer management. However, this treatment approach has various limitations, including the lack of tumor targeting by conventional chemotherapeutic drugs, which can lead to collateral damage to other organs or tissues in the body, and the potential development of drug resistance in tumor cells owing to prolonged exposure to high doses of a single antitumor agent. Consequently, there is a pressing demand for more effective and safer antitumor therapeutic strategies. Recently, researchers have conducted thorough investigations into innovative anticancer medications with a specific focus on composite drug delivery platforms utilizing nanomaterials. Notably, GO has emerged as a highly promising candidate for medical applications owing to its adjustable physicochemical characteristics, favorable biocompatibility, and convenient availability.

GO is a widely used oxidized form of graphene, which typically consists of single‐, bilayer, or multilayer graphene sheets with reactive functional groups such as hydroxyl (–OH), alkoxy (C–O–C), carbonyl (C–O), carboxylic acid (–COOH), and other oxygen‐based functional groups.^2^ GO possesses properties such as physical toxicity, genotoxicity, and strong near‐infrared absorption, which have been proven to cause significant killing of tumor cells.^3^ Exposure to 10–20 mg/L GO for 1 h induced cells to produce large amounts of ROS.^4^ This discovery inspired researchers to utilize GO to induce oxidative stress in tumor cells, thereby triggering significant photothermal and photodynamic effects, ultimately leading to a tumor‐killing effect. Notably, the large surface area of GO and the variety of highly active functional groups provide numerous binding sites. Therefore, covalent and noncovalent surface modification techniques can be used to achieve high‐density biofunctionalization and enhance the biocompatibility, dispersion, and cell adhesion properties of GO‐based composite nanomaterials. Additionally, functionalized GO can be employed to construct various drug carriers, enabling the controlled release of antitumor drugs, enhancing biofilm permeability, improving the bioavailability of orally administered drugs, and yielding various other beneficial effects.^5^ A growing body of research has employed GO to load targeting ligands,^6^ imaging probes,^7^ or to evade lysosomal degradation for controlled drug release,^8^ thereby endowing drug delivery systems with more diverse and efficient antitumor effects. Multiple approaches have conferred richer and more efficient antitumor effects on drug delivery systems. Recently, GO has also been utilized for capturing circulating tumor cells (CTCs), constructing biosensor platforms, tumor immunotherapy, and various other innovative areas of tumor therapy owing to its exceptional physicochemical properties. However, several challenges remain regarding its clinical translation, particularly the lack of standardized production methods, which leads to inconsistent therapeutic outcomes and potential biosafety risks. Studies have shown that variations in synthesis protocols across laboratories result in differences in the particle size and oxidation level of GO, potentially altering its biological effects.^9^ Moreover, GO can enter biological systems and may trigger adverse responses such as oxidative stress,^10^ tissue inflammation,^11^ and hemolysis.^12^ Further enhancement of the therapeutic effect while reducing damage to normal tissues remains one of the main focuses in the design of antitumor strategies.

The review further addresses the challenges associated with large‐scale production and biocompatibility during the clinical translation of GO, and proposes corresponding strategies for improvement. It aims to offer a theoretical and technical foundation for the safer and more efficient development of GO‐based cancer therapies, facilitating its broader application in clinical oncology.

## 2 INTRINSIC ANTITUMOR MECHANISMS OF PRISTINE GO

**FIGURE 1:**
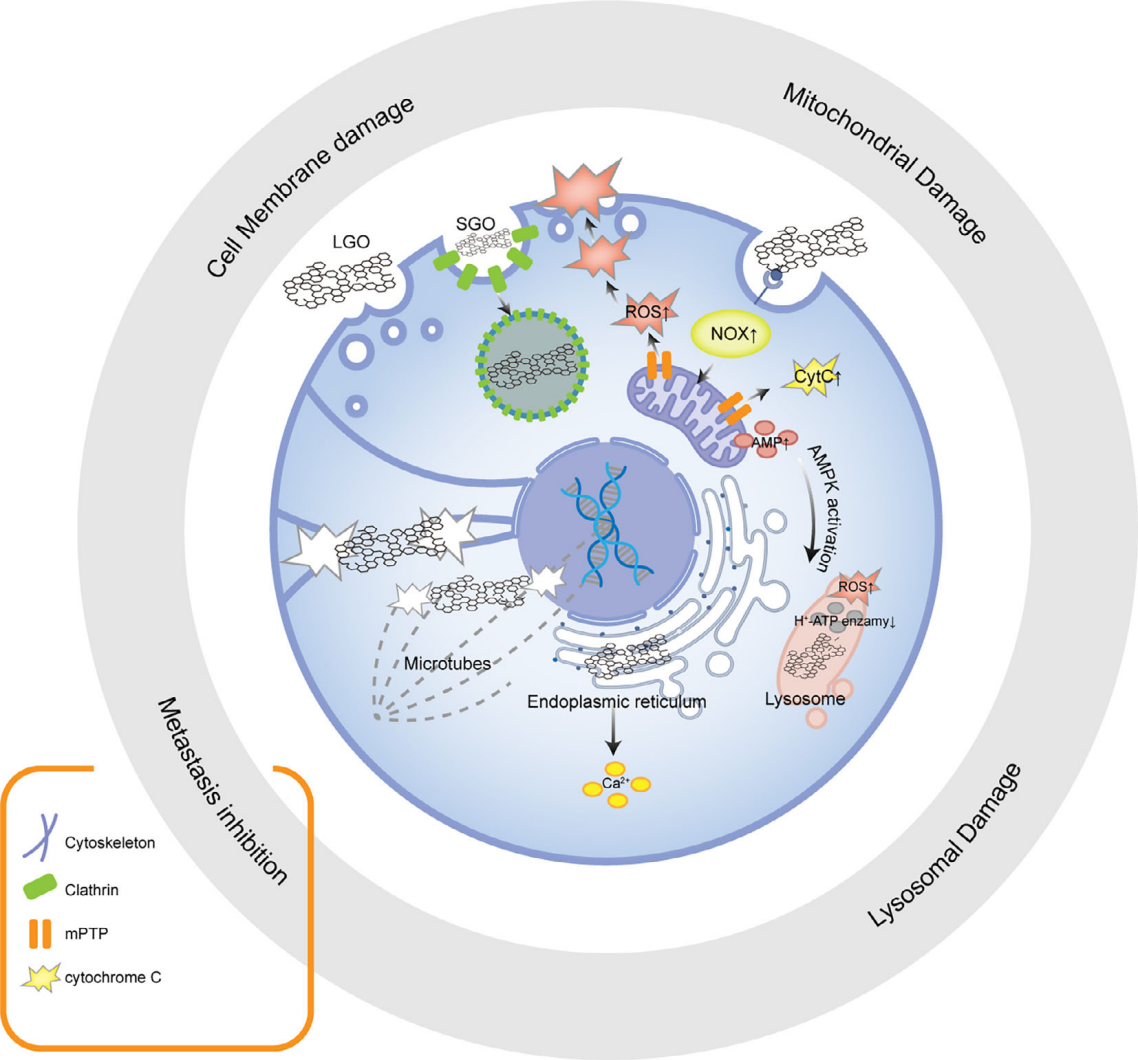
GO exerts potent antitumor effects through multimodal cellular damage: (1) Cell membrane disruption via direct physical insertion, electrostatic interactions or lipid peroxidation, compromising membrane integrity; (2) Lysosomal damage through destabilization of lysosomal membranes, increasing NOX family, H+ leakage and lysosome‐dependent cell death; (3) Mitochondrial dysfunction by inducing membrane potential collapse, ROS overproduction, and cytochrome c‐mediated apoptosis, upregulation of AMP activate AMPK pathway which further destroy Lysosome; (4) Cytoskeletal disruption by inhibiting filament bundling and disaggregating microtubes. These synergistic mechanisms collectively promote tumor cell death.

GO has emerged as a multifaceted agent in composite nanomedicine, exerting potent antitumor effects through direct physical and biochemical interactions with cancer cells. As illustrated in Figure 1, GO induces multimodal damage spanning from the plasma membrane, subcellular organelles (lysosomes, mitochondria, and endoplasmic reticulum) to the cytoskeleton, thereby disrupting tumor growth and proliferation. With outstanding nanosheet morphology, surface functional groups, and photothermal responsiveness, GO is a powerful platform for synergizing diverse anti‐cancer therapies to enhance therapeutic efficacy. This section systematically reviews the intrinsic antitumor mechanisms of pristine GO with a particular emphasis on its physicochemical determinants, thereby laying the groundwork for the rational design of synergistic therapeutic modalities integrating GO with auxiliary pharmaceutical agents Table 1.

**TABLE 1:** Antitumor Effects of GO in composite nanomedicine.

GO and GO‐based nanosystems	Applications	Size of GO/complexes	Cell line	Biological effects	References
GO	Membrane damage	/	U87, HeLa	Make pores on membrane and altering permeability, allow additional cisplatin inflow into the cells, resulte in cell death	[14]
GO	Mitochondrial damage	Lateral size:300‐800 nm	SH‐SY5Y	Trigger lipid peroxidation of the plasma membrane, induce ROS elevation by activating the NOX2 pathway, resulte in mitochondrial apoptosis through AMPK/mTORC1/ULK1 pathway	[18]
GRO‐NL		/	HT‐29, MCF‐7, Hela	Elevate intracellular ROS, which simultaneously altered ΔΨm, cause Ca^2+^ influx, result in the depletion of P53 protein, hamper the cell cycle checkpoints	[17]
GO	Proteasome inhibition	Lateral size: 0.5–3 μm Thickness:1 nm	4 T1	Induce the inhibition of the degradation of cell cycle regulatory protein p21 through suppressing the activity of 20S proteasome	[22]
PEG‐GO	Anti‐metastasis	Lateral size: 90 or 190 nm Thickness: 1.8 nm	Saos‐2	Impair F‐Actin filaments, inducing G0/G1 arrest, ROS accumulation and apoptosis	[23]
GO		Lateral size: 100‐200 nm Thickness: 3‐4 nm	Hela	Disrupts the actin cytoskeleton, prevent filament bundling and suppress invasion	[29]
PCL/BSA‐GO		Lateral size: 494 ± 65 nm	PANC‐1	Alter ECM topology, upregulate E‐cadherin and promotes keratin assembly, suppres migration	[25]
GO		Lateral size: 2–8 nm	U87, U118	Reduce phospho‐EGFR levels, increase *β*‐catenin/E‐cadherin binding, and inhibit EGFR/AKT/mTOR and Wnt/*β*‐catenin pathways, impairing invasion	[27]
GO	Dendritic cells activation	Lateral size: 225 ± 55 nm Thickness: 5.05 ± 1.35 nm	DC	Up‐regulate CD83, CD80, Tbet and FoxP3 induce inflammation in DCs, DC maturation, and potentially DC‐mediated T cell polarization	[32]
GO		Lateral size: 500 nm	DC	LGO induce TNF‐*α*, IL‐6 and NO upregulation, deliver antigen though TCR pathway and activate CD8T cell, enhance IFN‐*γ* and granzyme B production	[33]
siRNA‐aRGO‐Dox	Targeted anti‐tumor drug delivery and M2 macrophage depletion	Lateral size: 150 nm	4 T1	Eliminate M2 TAM, hold back Snail‐enhancing TGF‐*β* signal pathway, inhibit tumor growth	[37]
GO‐APP3/28	Adaptive immune activation	Lateral size: 5–80 nm	Raji	Increase antigen‐T cell contact area, enhance antigen delivery, activate T cell through TCR pathway, up‐regulate IL‐2 expression, increase CD4T differentiation	[39]

### 2.1 Direct cytotoxicity against tumor cells

GO damages cell membranes primarily through its physicochemical properties, molecular size, and interaction with membrane components. Kregielewski et al.^13^ showed that GO selectively disrupts cancer cell membranes at low concentrations via electrostatic and hydrophobic interactions with their abnormal membrane potentials, altering permeability and forming pores that directly induce cell death.

Studies have shown that GO promotes the generation of ROS by upregulating the expression of NOX1 and NOX2. This leads to an intracellular redox imbalance^14^ and concurrent damage to the cell membrane.^15^ Furthermore, under conditions of GO‐induced ROS overload and hypoxic stress, the mitochondrial permeability transition pore (mPTP) remains persistently open, which in turn can lead to two outcomes: the concentration‐dependent activation of the CytC‐Bax‐Caspase apoptotic pathway^16^, ^17^, and the stimulation of AMPK‐mediated autophagy through the accumulation of AMP.^18^ In addition, the sharp edges and steric hindrance of GO can disrupt the lysosomal membrane, leading to the leakage of hydrolytic enzymes^19^ and lysosomal alkalinization.^20^ Alternatively, it can impair the process of lysosomal acidification through AMPK‐mediated inhibition of v‐ATPase.^18^ Recent studies have revealed that GO dysregulates transcription factor EB (TFEB) and its upstream regulator STUB1, disrupting the autophagic‐lysosomal pathway (ALP) function and promoting apoptosis.^21^

Besides, Ma et al. found that large GO (0.5–3 μm) at low concentrations (10 μg/mL) blocks the *α*‐subunit entry of 20S proteasomes, inhibiting proteolytic activity and inducing G1‐phase arrest in breast cancer cells.^22^ Compared to mitochondria and lysosomes, GO's effects on other organelles remain understudied and warrant further exploration.

The multi‐level damage induced by GO, spanning from the plasma membrane to subcellular organelles (e.g., lysosomes, mitochondria, and endoplasmic reticulum), is illustrated in Figure 1, which underscores its multifunctional antitumor effect.

### 2.2 Suppression of tumor cell Invasion

The invasion and metastasis of tumor cells, as the basis for the difficulty in radical cure and recurrence of cancer, represent one of the hallmarks of advanced tumor progression. This process is closely associated with cytoskeletal remodeling, alterations in the extracellular matrix (ECM), the influence of the tumor microenvironment, and the interplay of related cellular pathways.

Research indicates that once internalized, GO localizes near F‐actin and disrupts the cytoskeleton through steric hindrance. This disruption inhibits the regulatory functions controlling the G1–S phase transition in osteosarcoma cells, thereby preventing tumor cell invasion.^23^ Additionally, GO can directly bind to tubulin, disrupting microtubule polymerization and depolymerization, thereby interfering with spindle formation and inducing cell cycle arrest.^24^ Moreover, by altering the composition and topology of the cell–matrix interface, GO promotes keratin assembly, suppresses epithelial–mesenchymal transition (EMT), and inhibits tumor cell migration.^25^

Beyond physical obstruction of the cytoskeleton, GO can impair mitochondrial respiration due to its higher electron affinity compared to iron–sulfur or iron‐porphyrin clusters in the mitochondrial ETC. By reducing the mitochondrial transmembrane potential, GO disrupts ATP supply for F‐actin assembly.^26^ GO nanoparticles also increase the affinity between *β*‐catenin and cadherin while downregulating the EGFR/AKT/mTOR and Wnt/*β*‐catenin signaling pathways in glioblastoma, thereby inhibiting tumor growth and invasion.^27^ Furthermore, GO significantly suppresses the expression of matrix metalloproteinases (MMPs) such as MMP2, MMP3, and MMP9 in the ECM in a dose‐dependent manner, preventing tumor cell invasion and metastasis induced by ECM degradation. It also downregulates metastasis‐related proteins, including intercellular adhesion molecule, vascular cell adhesion molecule, and collagen types I and III.^28^, ^29^

### 2.3 Enhancement of antitumor immunity

In recent years, immunotherapy has emerged as a hotspot in cancer research. GO activates innate immunity, adaptive immunity, and modulates the tumor microenvironment by promoting antigen presentation by antigen‐presenting cells (APCs), delivering target antigens, and stimulating receptor signaling in target cells, thereby enhancing immune surveillance and tumor elimination.^30^, ^31^

Numerous studies have demonstrated that GO exerts varying biological effects on innate immune cells, including macrophages, dendritic cells (DCs), natural killer cells (NKs), and neutrophils. Under GO culture conditions, the expression levels of activated DC biomarkers (CD83, CD80) are significantly elevated, indicating its broad immunostimulatory effects on DCs across different nanoparticle sizes.^32^ Parker et al. found that SGO tends to be internalized by DCs, preferentially activating CD4+ T cells, whereas LGO adsorbed on the cell membrane enhances DC‐induced CD8+ T cell activation. This difference may arise from distinct antigen cross‐presentation pathways depending on GO size and localization.^33^

GO also activates macrophages. Multiple studies have shown that GO of varying sizes (nano‐ and microscale) induces macrophage and neutrophil recruitment, triggering innate immune responses.^34^, ^35^ LGO activates Toll‐like receptor 4 (TLR‐4) on macrophage membranes and induces ROS generation to stimulate the NF‐κB pathway, synergistically promoting M1 polarization.^36^ Since M2 macrophages promote tumorigenesis and metastasis via the EMT transcription factor Snail/TGF‐*β* pathway,^37^ shifting macrophage polarization toward the M1 phenotype and reducing the M2/M1 ratio may represent a novel multidimensional strategy to simultaneously target tumors and M2 tumor‐associated macrophages (TAMs). For invariant natural killer T (iNKT) cells, GO polarizes them toward TGF‐*β* production and reduces pro‐inflammatory cytokines (IL‐4/IFN‐*γ*), expanding regulatory T (Treg) cells and attenuating iNKT‐mediated immunity.^38^

In adaptive immunity, Parker et al. reported that GO of all sizes significantly increased the co‐stimulatory molecule CD83 and upregulated T‐cell transcription factors T‐bet and FoxP3, skewing differentiation toward Th1.^33^ In the classical TCR activation pathway, GO‐mimicked APC platforms enhance T‐cell expansion efficiency. The α‐CD3/α‐CD28/GO platform stimulates potent IL‐2 autocrine signaling, overcoming the need for exogenous IL‐2 supplementation in CAR‐T therapy. It promotes CD4+ T‐cell differentiation and upregulates helper T and CD8+ T‐cell populations, demonstrating sustained tumor‐killing efficacy in human lymphoma and pancreatic cancer models.^39^ Furthermore, both GO and GONH2 activated naïve B cells, memory B cells, and plasma cells, inducing granzyme B (GrB) release and cytotoxicity against HeLa cells. Notably, GONH2 specifically upregulates CD38 and CD138, driving B‐cell‐to‐plasma‐cell differentiation and promoting tumor‐specific antibody production.^40^

Due to its strong immunostimulatory effects and excellent antibody‐delivery capacity, GO is an ideal adjuvant for enhancing vaccine efficacy. Its applications in tumor vaccines are detailed in the tumor prevention section.

## 3 GO ENGINEERED NANOCARRIERS FOR AUGMENTED EFFICACY

Owing to its large specific surface area and abundance of reactive functional groups, GO can be functionalized through both covalent and noncovalent modifications to construct multifunctional drug delivery systems. These platforms can co‐deliver multiple therapeutic agents to enhance synergistic anticancer effects while simultaneously incorporating various bioactive molecules to significantly improve biocompatibility, targeting specificity, and controlled‐release behavior. In this section, we summarize various GO‐based drug delivery systems designed to transport advanced chemotherapeutic agents and diverse bioactive molecules Table 2. By integrating photothermal therapy, chemotherapy, and targeted therapy, these systems establish multimodal synergistic treatment strategies, offering innovative solutions to overcome tumor heterogeneity and multidrug resistance.

**TABLE 2:** Design strategy and molecular mehanism of GO‐based nanoplatform in antitumor fields.

GO and GO‐based nanosystems	Applications	Size of GO/ complexes	Cell line	Biological effects	References
GO‐PEI‐PEG‐CPP/siRNA	Gene delivery	Size: 0.06–2 μm/−200 nm	MDA‐MB‐231	Enhance siRNA stability, promote cellular uptake, and improve siRNA delivery efficiency	[44]
GO‐PEI‐PSS/ADR/si‐miR21	Chemo‐gene co‐delivery	Lateral size: 50–300/500 ± 45 nm; thickness:1.2/1.57 nm	MCF‐7/ADR	Enhance drug concentration via the caveolae and clathrin‐mediated endocytosis pathways; downregulate miR21 to limit drug efflux function	[57]
GO‐PEG‐DTX	Sustained drug delivery	Lateral size: −/14.83 mm	DU‐145	Enhancement of drug concentration in tumor cells through permeability and retention effect	[41]
pGO‐FA‐PTX	Target tumor and antitumor drug delivery	Lateral size: 50–250/40–200 nm, thickness: 1.2/1.6 nm	A2780	Enter cells and locate in the nuclei with high efficiency, due to high affinity between FA and FA receptor overexpression in cancer cells	[50]
GO‐HA‐DOX		Lateral size: 100–200/350–950 nm	BT‐474 and MDA‐MB‐231	Enable better targeted drug delivery and increase cellular uptake	[51]
‐NGOPEGHN1‐DOX		Lateral size: 100–200/350–950 nm	CAL‐27SCC‐25	Enable particular target transport and effectual cell inhibition; pH‐responsive drug discharge features	[52]
GO‐Q		Thickness:3 ± 0.5/7.5 ± 1 nm	U87 tumor cells	By enhancing the cytotoxicity and cell cycle arrest effects of the drug in cancer cells, the anticancer efficacy is significantly improved.	[50]
GO‐HA‐RGD‐DOX	Targeted and sustained drug delivery	Lateral size: 70–490 nm, thickness: 1.2/13 nm	SKOV3	Using dual receptor targeting drug delivery system to enhance selectivity and targeted efficiency to cancer cells	[54]
APT‐GO‐CO‐γ‐PGA‐DOX	Target tumor and gene delivery	Lateral size:−/319.1 nm	Hela	Enable nucleus‐targeting effect, improve the targeting efficiency	[53]
GO‐PEI‐PEG‐CPP/si‐Rictor		Lateral size:195.2 ± 2.185/231.6 ± 3.261 nm	TNBC and MDA‐MB‐231	Enhance the cellular uptake and targeting ability; induce tumor apoptosis by interrupting PI3K/Akt/mTOR signaling	[44]
GO‐CS	Target and sustained gene delivery		Saos‐2 and MG‐63 osteosarcoma cells	Release siRNA in a pH‐dependent manner, demonstrate significant efficacy particularly in the acidic environment of tumor cells, while the material exhibits excellent biocompatibility and low inflammatory response.	[46]
GO‐PEG‐FA‐ICG	Targeted photothermal therapy	Lateral size 1.2/600 μm thickness: 2.5/20 nm		Perform simultaneously in vivo fluorescence diagnostic as well as combined PDT‐PTT effects for cancer treatments	[72]
GO@SiO_2_@AuNS	Targeted and sustained photothermal therapy	Lateral size: 1.0/300 μm	KM12C, SW620	Enables efficient photothermal conversion and photothermal treatment of tumor cells with excellent biocompatibility and photothermal stability	[67]
GO‐MB/PF127		Lateral size:62.9/ 121.8 nm	SiHa	The synergistic effect of simultaneous photothermal and photodynamic therapy produces a strong killing effect on tumor cells at low drug doses and kills SiHa cells via the apoptotic pathway	[70]
siSnailaRGODox	Targeted and sustained chemo‐gene co‐delivery with photothermal therapy	Lateral size: −/150 nm	4T1 cells	Targeted delivery and ROS‐responsive release of siRNA and DOX, inducing tumor cell apoptosis and silencing the Snail gene to prevent tumor cell migration and invasion.	[37]
GO/CS/IO microspheres	Targeting and controlled Drug Delivery with Magnetic Hyperthermia Therapy	Lateral size: −/623.99 nm	A172, T98, and L929	Enhancing therapeutic efficacy in glioblastoma treatment through pH‐triggered drug release and magnetic field‐assisted targeting.	[47]
GO‐PEG‐OSA‐PTX	Combat drug resistance	Lateral size: 103 ± 2.2/122 ± 2.0	PTX‐resistant GC (HGC‐27/PTX)	Possess pH/thermal‐sensitive drug release properties; Increase intracellular ROS levels to limit the efflux pump function of P‐gp	[56]

### 3.1 Augmentation of drug delivery fidelity

One of the advantages of using GO‐based drug carriers to deliver antitumor drugs is that they can overcome the solubility and stability problems of anticancer drugs, thus improving their antitumor effect. Many natural compounds used in traditional Chinese medicine, such as oridonin (ORI), exhibit significant hydrophobicity because of the absence of sufficient polar groups in their molecular structures. Zhang et al.^5^ employed polyethyleneimine (PEI)‐modified GO (PEI‐GO) to load ORI, which markedly enhanced its aqueous solubility, thereby improving drug delivery efficiency, saturation concentration, and therapeutic efficacy. In addition, PEGylated GO (GO‐PEG) can form stable nanocarriers through carboxyl–amino coupling reactions, significantly increasing the water solubility of the anticancer agent docetaxel (DTX) and effectively inducing cytotoxicity in prostate cancer cells.^41^ Furthermore, taking advantage of the huge specific surface area of GO, GONPs enable the co‐delivery of two drugs, quercetin (Qn) and lurbinectedin (Ln), via physical drug‐loading interactions, exhibiting significant cytotoxic effects and inducing apoptotic cellular death against lung cancer cell lines such as A549 and PC9.^42^

Nucleic acid‐based therapeutics have demonstrated significant potential for precision cancer treatment. However, their negative charge often leads to electrostatic repulsion with the cell membrane, limiting cellular uptake. This issue can be effectively addressed by modifying GO with cationic materials such as PEI, which facilitates nucleic acid loading and enhances cellular internalization, thereby improving bioavailability.^43^ Another major challenge in nucleic acid therapy is the vulnerability of RNA to intracellular enzymatic degradation, which results in reduced local efficacy. The steric hindrance effect of the GO lattice provides protective shielding against nucleic acids, thereby enhancing their stability and delivery efficiency. GO‐PEI‐PEG‐CPP nanoparticles have been shown to significantly improve the cellular uptake and delivery efficiency of siRictor, leading to apoptosis in breast cancer cells.^44^

In addition to polymer‐based modifications, metal oxides are also commonly employed for the surface functionalization of GO. For example, while curcumin (CUR) exhibits poor solubility in neutral or acidic tumor microenvironments, Matiyani et al.^45^ developed a TiO_2_@ZnO‐GO composite by leveraging the solubility properties of ZnO and the mesoporous structure of TiO_2_, which significantly increased the overall surface positive charge. This system co‐loaded CUR and quercetin, achieved sustained release under varying pH conditions, and further improved the drug loading capacity, stability, and targeting efficiency.

### 3.2 Endowing sustained drug release

Currently, the clinical application of nanomedicines remains limited by issues such as nonspecific drug interactions, multidrug resistance, and ATP‐driven drug efflux. As a highly tunable nanocarrier, GO can be engineered to respond to specific stimuli for controlled drug release, thereby ensuring precise delivery, enhancing cellular uptake, and promoting intracellular accumulation.

PH‐triggered drug release has emerged as one of the most commonly employed strategies for anticancer drug delivery systems. GO functionalized with the cationic polymer chitosan (CS) can efficiently adsorb Bcl‐2 siRNA through electrostatic interactions. In the mildly acidic tumor microenvironment, the ionic interactions between CS and Bcl‐2 siRNA are weakened, allowing for targeted release of siRNA from the GO‐CS complex and inducing apoptosis in osteosarcoma cells.^8^ Additionally, Li et al.^46^ developed a dual‐targeting delivery system by modifying GO with HA and HN‐1 to co‐deliver DOX. The protonation of DOX amine groups under acidic conditions enhances hydrophilicity and weakens hydrogen bonding with GO, leading to a cumulative drug release 3.3 times higher than that under neutral conditions. Beyond pH and NIR responsiveness, magnetic molecules have also been explored for controlled drug release. A magnetic microsphere system composed of Fe_3_O_4_ (IO), GO, and CS for temozolomide (TMZ) delivery was developed, which, under a 100 Hz alternating magnetic field, not only enabled efficient drug release but also achieved precise targeting through magnetic‐field guidance and promoted intracellular transport of magnetic nanoparticles, thereby further enhancing the therapeutic efficacy and targeting specificity of the system.^47^ ROS responsiveness has also emerged as a novel drug release mechanism. Wang et al.^37^ demonstrated that ROS‐sensitive molecules (RCSP) could be closely integrated with GO via electrostatic adsorption and boronic ester crosslinking to form an ROS‐responsive delivery system. Upon internalization by tumor cells and M2 TAMs, elevated ROS levels induce oxidative degradation of RCSP and cleavage of the crosslinking bonds, thereby triggering the release of siSnail and DOX.

### 3.3 Augmented tumor‐trgeted delivery

As a pivotal strategy in current antitumor therapies, targeted drug delivery can significantly enhance intratumoral drug accumulation, thereby improving therapeutic efficacy while minimizing off‐target toxicity to normal tissues. GO‐based targeted delivery systems not only enable high drug‐loading capacity and controlled release but, through functionalization with targeting ligands, also achieve selective accumulation in tumor cells, ultimately improving overall treatment outcomes.

Owing to the enhanced permeability and retention (EPR) effect, GO with a relatively small size (e.g., ~50 nm) can achieve high tumor accumulation via passive targeting. For instance, Zygouri et al.^48^ loaded quercetin onto GO (GO‐Q) and leveraged its nanoscale dimensions to enhance EPR‐mediated accumulation within tumor tissues. However, such accumulation may diminish over time due to systemic clearance mechanisms.^49^

Beyond passive targeting, increasing research has focused on endowing GO with active targeting capabilities by conjugating it with specific ligands. For instance, folic acid (FA) can form covalent bonds between its hydroxyl groups and the carboxyl groups on GO, yielding a GO‐FA–paclitaxel (PTX) delivery system that actively targets folate receptor‐overexpressing colorectal and ovarian cancer cells.^50^ Similarly, hyaluronic acid (HA), known for its specific binding affinity for CD44 receptors, can be covalently conjugated to GO and loaded with DOX to selectively eliminate CD44‐positive breast cancer cells.^51^ Moreover, the tumor‐targeting peptide HN‐1 can be covalently coupled to nGO‐PEG via hydrogen bonding and π–π stacking to construct the nGO‐PEG‐HN‐1 nanocarrier, which significantly enhances cytotoxicity against oral squamous cell carcinoma (OSCC) while reducing off‐target toxicity.^52^ Another strategy involves grafting the nucleolin‐targeting aptamer NH₂‐AS1411 (APT) onto GO–chitosan (CO)–γ‐polyglutamic acid (PGA) composites, forming an APT‐functionalized delivery system that promotes DOX uptake in nucleolin‐overexpressing HeLa cells while minimizing cytotoxicity in normal pulmonary epithelial cells.^53^

**FIGURE 2:**
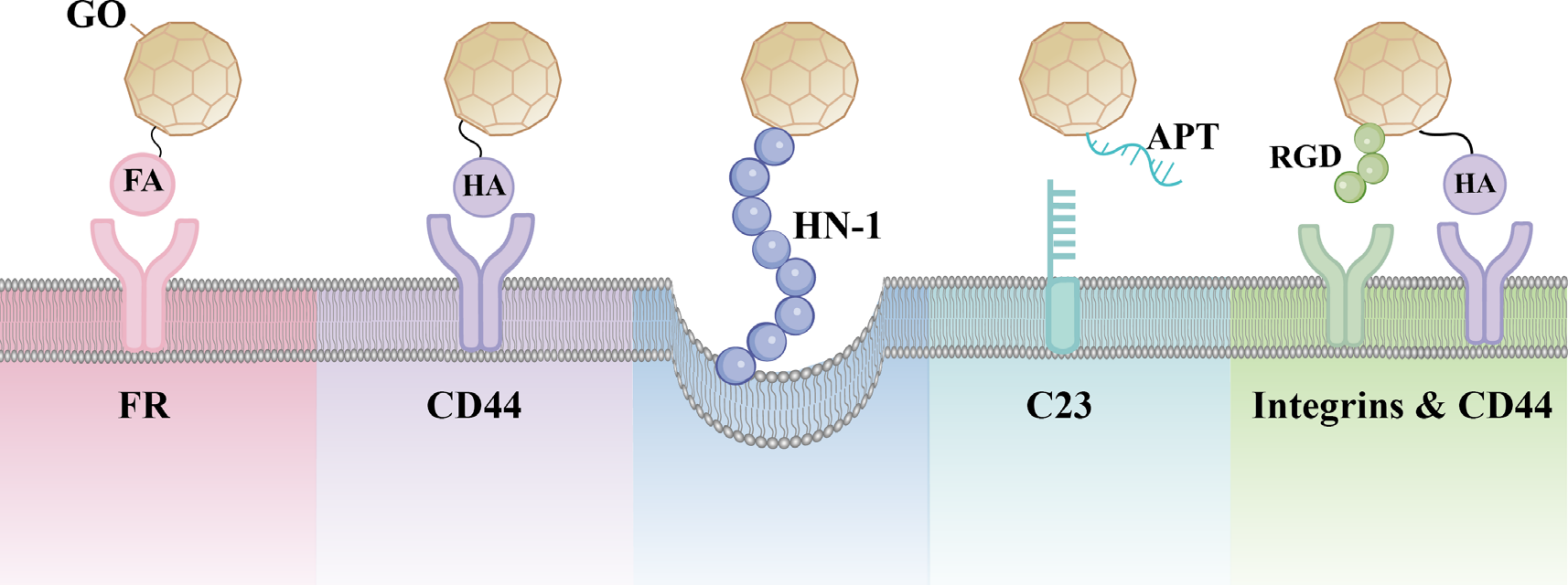
The illustration depicts GO‐based targeted drug delivery systems actively targeting tumor cells through different molecular targets, arranged from left to right as follows: (1) Folic acid (FA) targeting folate receptors (FR) overexpressed on colorectal and ovarian cancer cell surfaces; (2) Hyaluronic acid (HA) targeting CD44 receptors on breast cancer cells; (3) Tumor‐targeting peptide HN‐1 specifically binding to oral squamous cell carcinoma; (4) Nucleolin‐targeting aptamer APT recognizing surface protein nucleolin (C23) on cervical cancer cells; (5) Dual targeting system where HA and RGD peptide simultaneously engage CD44 receptors and integrins on tumor cell surfaces respectively.

In addition to single‐ligand targeting strategies, the development of multitargeting GO‐based platforms has attracted considerable attention. For example, GO drug carriers co‐functionalized with HA and RGD peptides demonstrated enhanced apoptotic effects in human ovarian cancer cells (SKOV3) when delivering DOX, compared to carriers with single‐ligand modifications.^54^ Notably, even with a modest decrease in drug‐loading capacity, dual‐targeting GO systems can still induce significant cytotoxicity, suggesting that the rational design of dual‐ or multi‐ligand functionalization may represent a promising avenue for future research. All the aforementioned GO‐based active targeting carriers are summarized and illustrated in Figure 2.

### 3.4 Overcoming tumor drug resistance

Tumor cell resistance is a fundamental reason for the limited efficacy of many anticancer drugs in later stages of treatment. Researchers have utilized GO‐based drug delivery systems to reverse tumor cell resistance based on different resistance mechanisms. Cisplatin (CDDP), a commonly used chemotherapeutic drug, easily forms complexes with glutathione (GSH) in tumor cells, preventing CDDP from entering the cell nucleus and leading to drug resistance. By employing manganese dioxide‐modified nGO (nGO@MH) as a carrier for CDDP, the chemical reaction between manganese dioxide and GSH in tumor cells reduces the consumption of CDDP before it enters the nucleus, effectively overcoming tumor cell resistance.^55^ A more effective method for reversing tumor cell resistance is to block the activity of the efflux protein P‐gp. Using GO‐based drug carriers with NIR radiation to inhibit P‐gp efflux function, a GO‐paclitaxel system can reduce drug efflux under NIR exposure, effectively suppressing drug resistance in gastric cancer cells.^56^ Additionally, it has been found that the upregulation of miR‐21 is associated with breast cancer resistance. To address this, researchers have developed a nano‐platform using PEI and sodium poly(4‐styrenesulfonate) (PSS) modified GO for the delivery of miR‐21 siRNA and doxorubicin (ADR), effectively reversing multidrug resistance,^57^ Therefore, GO, through its own mechanisms or by delivering drug‐resistant molecules, effectively reverses drug resistance, offering a promising approach to synergistically enhance the therapeutic effects of anticancer drugs.

### 3.5 Nanocarriers of tumor profiling

Due to the π‐electrons in the localized sp^2^ conjugated structure of GO, weak fluorescence can be emitted under specific wavelength excitation (e.g., 400 nm visible light or 658 nm near‐infrared light).^58^ When combined with fluorophores, radionuclides, and other compounds, GO can be used to develop tumor therapy platforms with both cancer imaging and treatment functions, thus enabling the visualization of anticancer drugs. Researchers conjugated GO polyethylene glycol (GOP) dual‐drug targeted nanocomplexes with fluorescein isothiocyanate (FITC), achieving intracellular FITC localization and real‐time fluorescent imaging of its accumulation.^59^ Additionally, GO can be loaded with other fluorescent molecules, such as rhodamine B115^60^ and silicon phthalocyanine (SiPc),^61^ which also reveal the accumulation and distribution of nanocomposites inside cells. Researchers have also replaced FITC with magnetic MRI for drug tracking and cellular imaging. For example, a composite drug delivery system designed using the interaction between GO and Fe_3_O_4_ enhances MRI signal strength, enabling in vivo drug tracking.^62^ Other magnetic particles, such as ultra‐small superparamagnetic iron oxide (USPIO), can also be combined with GO for magnetic resonance imaging.^63^

Photoacoustic imaging (PAI), which integrates optical and ultrasound (US) detection, offers superior contrast and spatial resolution compared to traditional purely optical imaging techniques.^64^ FA conjugated with chitosan (CS) to form the targeted contrast agent FA‐CS‐GO demonstrates high imaging efficiency in vivo, as shown by strong photoacoustic signals following tail vein injection in mice.^65^ By combining this imaging method with GO proprietary photothermal therapy, tumor imaging and treatment can be performed simultaneously.

### 3.6 Synergy‐enhanced photoresponsivity

The unique physical characteristics of GO, including its large surface area and oxygen‐containing functional groups, enable it to serve as a versatile material for photothermal or photodynamic applications, while also facilitating the attachment of other functional molecules. GO is a highly favored photosensitizer carrier in photodynamic therapy (PDT).^66^ In addition, silica‐modified GO can provide abundant binding sites for gold nanoparticles, forming zero‐dimensional gold nanoshells that significantly enhance the photothermal effects of nanogold.^67^ Moreover, since the hypoxic environment within tumors can limit the efficacy of photosensitizers, GO‐based systems can co‐deliver manganese oxide (MnO) to alleviate hypoxia and ensure sustained ROS generation at cytotoxic levels, thereby enhancing the therapeutic outcome of PDT.^68^ By modifying GO with the photosensitizer Protoporphyrin IX (PPIX) and loading it with the chemotherapy drug Osimertinib (AZD) and targeting agent HA, Zhang et al. designed a multifunctional platform capable of delivering a synergistic combination of chemotherapy, photothermal, and photodynamic therapies.^69^ Additionally, other photosensitizers—such as methylene blue (MB),^70^ indocyanine green (ICG),^71^, ^72^ and chlorin e6 (Ce6)^66^ can also be combined with GO to enhance both photostability and the overall efficacy of photothermal and photodynamic therapies.

Furthermore, the photothermal effect of GO has been explored in combination with other cytotoxic effects. In Huang's study, GO was used to co‐deliver a DNA oxidation repair enzyme inhibitor and a photosensitizer, thereby enhancing ROS sensitivity and triggering apoptosis.^73^ Itoo et al.^74^ utilized PEG‐modified GO loaded with oxaliplatin (OX) and platinum (IV) prodrugs. While inducing DNA damage and apoptosis to directly kill tumor cells, GO also generates localized high temperatures under near‐infrared laser irradiation, causing thermal damage and cell death, thereby synergistically enhancing the chemotherapy effect. By co‐modifying GO with the targeting agent HA, the photosensitizer Ce6, and gold nanorods, a single nanocarrier was developed to integrate targeting, diagnosis, chemotherapy, PTT, and PDT. This system efficiently inhibited the growth of murine cervical cancer in mice through synergistic effects.^75^

## 4 NOVEL APPLICATIONS OF GO IN TUMOR PREVENTION AND MONITORING

Owing to its distinctive physicochemical properties, graphene oxide (GO) exhibits broad application prospects in the realm of cancer prevention and surveillance. Through the engineering of functionalized nanoplatforms, GO significantly enhances the capture and release of tumor‐associated biomarkers while amplifying signal transduction fidelity, thereby improving the sensitivity and specificity of tumor monitoring. This advancement provides novel strategies for early cancer diagnosis, dynamic therapeutic tracking, and precision efficacy evaluation. Furthermore, GO's dual functionality as both a nanovector and immunoadjuvant potentiates antigen delivery efficiency, activates lymphocyte populations, and promotes sustained antitumor immune memory, demonstrating remarkable potential in next‐generation tumor vaccine development.

### 4.1 Tumor surveillance platforms

Liquid biopsy is an emerging cancer screening method that analyzes biomarkers, such as CTCs, circulating microRNAs, circulating tumor DNA (ctDNA), and exosomes, in bodily fluids, providing guidance for early cancer diagnosis, tumor metastasis detection, and post‐operative tumor monitoring. GO possesses an excellent surface area, superior biocompatibility, abundant binding sites, and unique electrical and thermal properties, enabling it to overcome limitations such as low blood concentrations of tumor markers, low recognition sensitivity, and inefficient post‐capture detection. These attributes make GO an ideal substrate for constructing a functionalized platform.

To address the challenge of releasing captured CTCs from substrates in detection methods, Yoon et al. combined GO with thermo‐responsive polymer N‐acryloylpiperidine‐co‐N and N‐diethylacrylamide (AP‐DEA), achieving a CTC release rate of up to 91%.^76^ MicroRNAs are excellent biomarkers for early cancer detection in blood. The surface of GO consists primarily of abundant aromatic and oxidized functional groups, which can serve as energy acceptors for fluorescence‐labeled duplex structures (e.g., FAM, AMC, and FITC), making it an effective photochemical platform. Nucleobases interact with the sp^2^‐hybridized aromatic domains of GO via π–π stacking, adsorbing onto the surface, and quenching fluorescence. In the presence of target molecules, the higher affinity between the target nucleic acid and the labeled probe displaces the nucleic acid complex from GO, restoring the biosensor's fluorescence signal and enabling quantitative detection.^77^ Studies have demonstrated that circulating miRNAs can be detected photochemically in gastric cancer (miRNA‐21),^78^ nasopharyngeal carcinoma (miRNA‐205),^79^ and prostate cancer (miRNA‐141),^80^ with detection limits reaching the picomolar (pM) level and both significantly amplified fluorescence signals.

Similarly, in clinical settings, polymerase chain reaction (PCR), a common method for detecting ctDNA, suffers from drawbacks such as increased background nucleotides, reducing sensitivity^81^ and interference from other tumor microenvironment components, lowering specificity.^82^ GO‐based field‐effect transistor biosensors convert the microcurrent signal generated by hybridization between target gene sequences and electrode‐surface probes into readable electrical signals, amplifying them to enhance the recognition of weak biological signals, achieving femtomolar (fM) sensitivity.^83^ Mahbubur Rahman et al. developed an electrochemical detection system using an electrochemically active rGO‐AuNPs sensor, modified with L‐arginine polymers to improve charge transfer efficiency, pushing the detection limit below the attomolar (aM) level.^84^ Zihni et al. utilized GO's abundant carbonyl groups to immobilize Cas9‐sgRNA complexes (CRISPR/dCas9), which stably bind ctDNA carrying tumor‐specific genes, achieving a 96% detection rate.^85^

Furthermore, conventional exosome‐specific probes often suffer from instability, high costs, and low sensitivity. Feng et al. employed molecularly imprinted polymers (MIPs) on GO as recognition elements, creating a sensor platform with lower reliance on target protein abundance, higher stability, and improved resistance to background interference.^86^ Gurunathan et al. demonstrated that GO induces oxidative stress in breast cancer cells, activating n‐sphingomyelinase to promote exosome biogenesis and release, thereby increasing exosome target concentration.^87^

**FIGURE 3:**
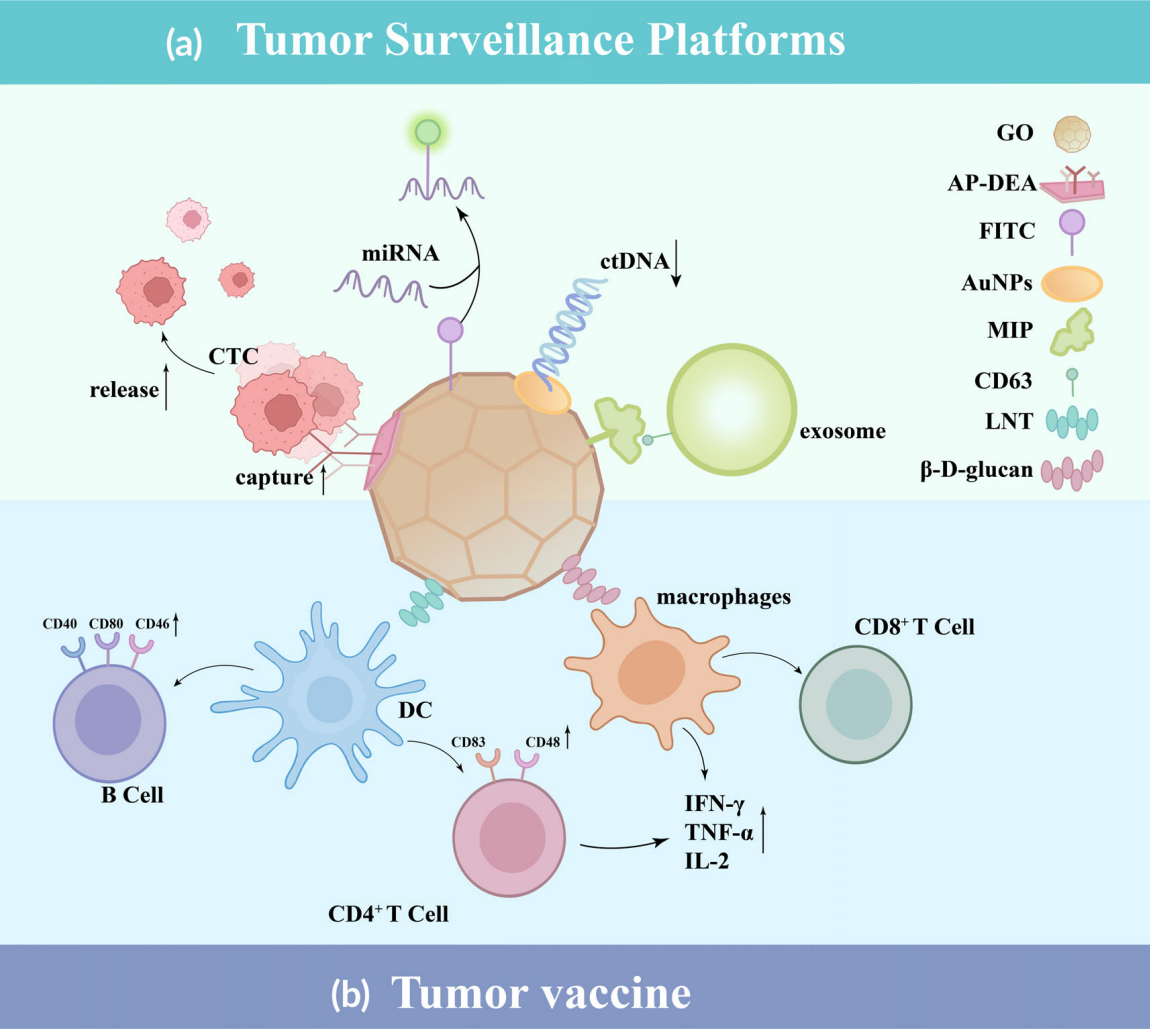
(a) Schematic diagrams of tumor monitoring platforms corresponding to different biomarkers: (1) Efficient in vivo capture and in vitro release of circulating tumor cells (CTCs) via AP‐DEA surface coating; (2) Fluorescence‐based in vivo quantification of miRNA leveraging its high affinity for FITC; (3) High‐efficiency recognition of ctDNA by loading advanced sensors (e.g., AuNPs) onto GO; (4) Enhanced exosome detection sensitivity using molecularly imprinted polymers (MIPs) as recognition elements. (b) Schematic illustration of GO‐mediated immune activation: (1) LNT‐GO activates dendritic cells (DCs), thereby enhancing B cell effector functions and activating CD4^+^T cells; (2) CpG‐loaded GO targets macrophages via *β*‐D‐glucan binding and promotes CD8+ T cell activation.

In summary, GO‐based biosensors have enhanced the sensitivity and specificity of biomarker detection through surface modification and functionalized component loading, as summarized in Figure 3a, demonstrating their broad prospects in tumor monitoring.

### 4.2 Tumor vaccine

In recent years, personalized tumor vaccines have emerged as a novel anti‐tumor strategy following immunotherapies, such as chimeric antigen receptor T‐cell (CAR‐T) therapy and immune checkpoint inhibitors (CPIs), guiding long‐term specific tumor immunity.^88^ GO‐based tumor vaccines can penetrate vesicle membranes, disrupt endosomal or lysosomal membrane stability, and promote antigen release, enhancing antigen presentation efficiency.^89^ Additionally, Yin et al. used polyethyleneimine (PEI)‐functionalized GO hydrogels to encapsulate ovalbumin mRNA (mOVA), leveraging electrostatic adsorption for sustained nucleic acid release.^90^

GO also acts as an adjuvant, enhancing immune responses by promoting antigen presentation, activating autophagy pathways, and inducing cytokine secretion. Lentinan‐functionalized GO hydrogel (LNT‐GO Gel) significantly upregulated CD40, CD80, and CD86 expression, while IgG levels showed an upward trend over 42 days, indicating GO's role in DCs maturation and B‐lymphocyte activation.^91^ *β*‐D‐glucan‐modified GO delivers Toll‐like receptor (TLR) agonist CpG oligodeoxynucleotides to antigen‐presenting cells (APCs), particularly macrophages, activating TLR9 and stimulating anti‐tumor cytokine secretion and CD8^+^ T‐cell activation.^92^ Beyond serving as an antigen carrier, GO itself enhances APC activation/recruitment and T‐cell proliferation. For instance, GO triggers the TLR4/TLR9‐NF‐κB pathway, promoting macrophage autophagy and downstream cytokine production (e.g., TNF‐*α* and IL‐1*β*).^93^ GO upregulates CD83 expression in DCs, inducing CD4^+^ T‐cell activation, which in turn secretes cytokines (e.g., IFN‐*γ*, TNF‐*α*, IL‐2) to further mature APCs, creating a positive feedback loop.^33^ In addition to being an immunomodulator, GO can also promote tumor immunotherapy through cell therapy engineering. For instance, GO loaded with CD3 and CD28 can achieve in vivo T‐cell activation and expansion, and can effectively stimulate T cells (especially CD4^+^ T cells) to autonomously secrete sufficient IL‐2.^39^

Notably, GO's interaction with APCs is size‐dependent. Large GO sheets promote DC–T‐cell cluster formation, upregulating CD48 and MHC I expression in DCs, and fostering a T‐cell‐activating microenvironment.^94^ In contrast, nano‐sized GO (85 nm) is internalized by APCs, exhibiting concentration‐dependent ROS reduction and chemotactic effects similar to tumor necrosis extracts, making it a safe vaccine delivery size.^95^

These studies highlight the advantages of GO as a versatile nanovaccine platform, including its tumor‐targeted delivery, sustained antigen release, and effective induction of specific cellular immunity, as summarized in Figure 3b. GO represents a promising carrier to overcome immunosuppression or tolerance within the tumor microenvironment, with potential therapeutic and preventive clinical applications.

## 5 CLINICAL TRANSLATION AND REGULATORY CONSIDERATIONS

The requirements set by the FDA for the clinical translation of nanodrugs include the capability for large‐scale manufacturing, stable product characteristics, and high biocompatibility.^96^ According to current research, the biocompatibility of GO is influenced by multiple factors, such as its particle size, degree of surface modification, dispersion level, and route of administration. Concurrently, the absence of standardized production methods leads to variations in the physicochemical properties of GO, which complicates a unified safety assessment.^97^, ^98^ These factors collectively pose significant hurdles to the clinical translation of GO as a drug carrier.

### 5.1 Current clinical studies of GO

To evaluate the challenges facing GO in clinical translation, we conducted a systematic search for GO‐related clinical studies across databases including the National Library of Medicine, ChiCTR, EU CTR, and the WHO ICTRP. This search identified only three relevant clinical studies, which included one randomized controlled trial.

Among these, Andrews et al. reported the first‐in‐human safety assessment of inhaled small‐sized (sGO) and ultra‐small‐sized (usGO) graphene oxide on the cardiorespiratory system. The study found no adverse reactions in healthy individuals following acute exposure to these high‐purity GO nanosheets, suggesting a favorable safety profile.^99^ In a separate application, Soundarajan et al.^100^ utilized GO for local oral therapy. By leveraging the high specific surface area and hydrophilicity of GO to load silver (Ag), they significantly enhanced the stability of the nanoparticles, thereby circumventing the side effects associated with chlorhexidine, a standard clinical mouthwash. Lastly, Mahshid Manouchehri et al. developed a highly efficient micro‐solid phase extraction (μSPE) technique by functionalizing GO with porphyrin, capitalizing on its unique surface properties. This method was designed for the separation and detection of non‐steroidal anti‐inflammatory drugs (NSAIDs) in urine samples.^101^ While these studies demonstrate the preliminary potential of GO in the biomedical field, its clinical translation remains in a nascent stage.

### 5.2 Production challenges in large‐scale translation

In 1958, the Hummers method first utilized potassium permanganate (KMnO_4_), sodium nitrate (NaNO_3_), and concentrated sulfuric acid (H_2_SO_4_) for the synthesis of GO.^102^ Subsequently, researchers have developed numerous more efficient and convenient synthesis methods, such as two‐step, nitrate‐free, co‐oxidant, and room‐ and low‐temperature approaches.^103^ However, modifications to the Hummers method vary between experiments, leading to instability in the molecular length, thickness, and degree of oxidation of the produced graphene. For instance, regarding the oxidizing agents, replacing NaNO_3_ with phosphoric acid (H_3_PO_4_)^104^ or partially substituting KMnO_4_ with K_2_FeO_4_ ^105^ can both significantly increase the degree of GO oxidation and promote the formation of more oxygen‐containing functional groups. Nevertheless, minor variations in the amount of oxidant used,^106^ mixing efficiency or purification,^107^ and reaction time and temperature^108^ can greatly impact the quality and oxidation degree of GO. Moreover, the modified Hummers method exhibits poor synthesis reproducibility. Different batches of GO present a random distribution of sizes and morphologies, ranging from nanoscale fragments to micrometer‐scale large sheets, while also containing varying concentrations of residual metal impurities.^109^ Additionally, post‐synthesis processing steps such as Base Washing, Sonication, and Cleaning all affect GO's chemical composition, including an increased C/O ratio, as well as its physical morphology (changing from planar to wrinkled) and size.^110^

Moreover, challenges related to the stability and storage of GO also present significant hurdles to its clinical translation. To meet transportation and storage requirements, the FDA mandates the commercial distribution of GO in powder form. However, powders prepared via conventional high‐pressure thermal drying often suffer from reduced specific surface area, sheet aggregation, and localized hydrogen bond rearrangement, significantly impairing dispersibility and functional activity.^111^ Freeze‐drying, though widely used, also poses challenges, as ice crystal compression can induce irreversible π–π stacking and hydrogen bonding between GO layers, hindering redispersion in aqueous media.^112^ To address these issues, Chen et al.^113^ developed an aerosol spray pyrolysis method to fabricate flower‐shaped GO (fGO) powders with a unique wrinkled morphology, effectively preventing sheet aggregation and improving both dispersibility and structural integrity. Dimiev et al.^114^ demonstrated that GO gradually releases H^+^ ions during prolonged water exposure, rendering hydroxyl groups increasingly acidic and altering the material's structural framework. Similarly, Kim et al.^115^ observed a spontaneous transformation from epoxide to hydroxyl groups at room temperature, stabilizing after approximately 35 days, which increases GO's hydrophilicity and biocompatibility.^116^ Whether this transformation persists after drug loading, however, remains to be elucidated.

The clinical translation of nanomaterials on a large scale is contingent upon adherence to robust manufacturing controls and the comprehensive characterization of their material components, as mandated by the FDA. (See also other FDA guidances for industry that establish recommendations for process improvement as manufacturing experience is gained; e.g., ICH Q10 Pharmaceutical Quality System (April 2009), and Process Validation.) The primary challenges for the large‐scale production of GO include the risk of thermal runaway, the controllability of the oxidation process, and the efficiency of purification.^9^ When synthesizing GO via the Hummers method, strict temperature control is crucial, as exceeding 55°C poses a risk of “thermal runaway” and even explosion.^117^, ^118^ Concurrently, the degree of oxidation in GO is co‐influenced by multiple factors, including the type and amount of oxidant,^106^ as well as reaction time and temperature, making precise control during large‐scale production difficult.^119^ Furthermore, in post‐processing, challenges persist in efficiently separating and purifying GO to remove impurities^120^ and in obtaining 2D nanosheets of uniform size and thickness,^121^ both of which are significant hurdles for this technology to advance to large‐scale production.

### 5.3 Biocompatibility assessment

A growing number of studies have revealed that GO can cause various forms of cell or issue damage, including hemolysis,^12^ oxidative stress,^10^ and tissue inflammation.^11^ Numerous variables, including GO exposure dose, size, and surface modification, affect its harmful effects. For instance, the percentage of cell viability of human prostate cancer DU145 was 75% after 48 h of incubation with 4 mg/mL GO; however, when the GO concentration was increased to 80 mg/mL, the percentage of cell viability dramatically dropped to 10%.^122^ The relationship between size and harmful consequences of GO can be even more nuanced. In vitro, Leydig cells (TM3) and Sertoli cells (TM4) produced higher levels of ROS when exposed to GO of a smaller size (20 nm) than when exposed to a larger size (100 nm) of GO.^123^ However, in mice, the hepatotoxicity of the smaller GO sheet was mitigated as it was more likely to be cleared via hepatobiliary excretion than the larger GO sheet.^124^ Additionally, through oropharyngeal aspiration, small particle size GO (10–700 nm) is phagocytosed by neutrophils in mouse lung tissues and degrades, while large particle size GO (0.5–2.5 μm), for which phagocytosis is frustrated, leads to a focal immune response in lung tissues.^125^ Different exposure routes elicited different toxic responses by influencing the biological distribution of GO within the body. For instance, when GO is injected intraperitoneally, it can be absorbed into the bloodstream, leading to an excess of small red blood cells and coagulation disorders in the blood of rats.^126^ When GO was orally administered to rats, it disrupted the microstructure of the small intestine through the ROS‐mitochondrial homeostasis‐apoptosis axis.^14^

GO has a high specific surface area and abundant chemical functional groups, making it easy to functionalize. The GO surface can be modified by altering oxygen‐containing groups and protein layers, among other methods, to enhance biosafety. The comet assay revealed that the DNA toxicity of hydroxylated GO in ARPE‐19 cells was significantly reduced compared to that of the group treated with GO alone,^127^ indicating that the incorporation of hydroxyl groups is an effective strategy for enhancing the biocompatibility of GO. Furthermore, GO can be surface modified to reduce its toxic effects. For example, the hemocompatibility of GO is significantly enhanced by PFG modification, which may be attributed to the formation of a barrier on the GO surface.^128^ Similarly, the application of a BSA coating on a GO surface reduced the physical disruption of cell membranes.^129^ One benefit of using GO as a drug carrier is its exceptionally high loading capacity. However, the biosafety profile of GO can be altered depending on the route of exposure.^130^ Consequently, the clinical translation of modified GO materials may also necessitate a thorough discussion of the impact of its administration route.

## 6 SUMMARY AND FUTURE PROSPECTS

Currently, chemotherapy remains a primary strategy in cancer treatment. However, its clinical effectiveness is often limited by several challenges, including poor targeting specificity, the development of drug resistance, and significant toxic side effects during the process of delivering drugs efficiently to tumor cells.

In recent years, nanomaterial‐based functional drug delivery systems have emerged as versatile platforms to overcome the limitations of chemotherapy and enable combinatorial therapeutic strategies. Graphene oxide (GO), as a novel nanoplatform for cancer treatment, not only enhances antitumor efficacy through mechanisms such as inducing oxidative stress, inhibiting tumor cell migration, and activating tumor‐specific immune responses, but also offers unique structural advantages. Its large specific surface area and abundance of oxygen‐containing functional groups provide multiple binding sites, facilitating the conjugation and surface modification with various biomolecules—including proteins, nucleic acids, and photosensitizers—for efficient loading and targeted delivery of multifunctional anticancer agents. In addition, GO's excellent biocompatibility and strong affinity for biological macromolecules make it a promising candidate for high‐sensitivity, noninvasive tumor monitoring platforms and for the development of safe and long‐lasting cancer vaccines.

This study, based on GO's distinct physicochemical properties and antitumor mechanisms, explores its potential applications and clinical translation pathways in oncology. On one hand, GO can be engineered via surface modification to serve as a versatile platform for loading a variety of agents, including targeting molecules, chemotherapeutic drugs, fluorescent markers, and cancer vaccines. Such a platform enhances the targeting specificity and therapeutic efficiency of drug delivery, improves capabilities for tumor imaging and monitoring, and ultimately aims to achieve comprehensive therapeutic goals such as direct tumor eradication and the inhibition of metastasis. On the other hand, the clinical application of GO‐based nanoplatforms is significantly impeded by key challenges. The lack of standardized production methods, coupled with the instability of the material post‐functionalization, currently precludes both scalable manufacturing and consistent biosafety assessments.

Delivery platforms such as liposomes^131^ and polymer nanoparticles^132^ which already include multiple FDA‐approved products, are well‐established platforms known for their high biocompatibility, biodegradability, ease of modification, and superior controlled‐release properties. However, their drug‐loading efficiency, limited by their intrinsic structures (e.g., the volume of the lipid bilayer and membrane stability, or the specific surface area of the polymer), is far inferior to that of GO. Conversely, inorganic nanoparticles like AuNPs and Fe_3_O_4_ are emerging as promising agents for thermal therapies and tumor imaging, although their biocompatibility warrants further investigation.^133^ By combining the advantages of both organic and inorganic materials, the GO platform exhibits broad prospects for cancer therapy. It is particularly well‐suited for developing synergistic therapies that combine multiple treatment modalities, including chemotherapy, thermal therapy, gene therapy, and imaging‐guided tracking. In the future, in‐depth research into standardizing production methods and refining application strategies could further advance its clinical translation and application in the field of antitumor therapy.

## AUTHOR CONTRIBUTIONS

**Xiaoli Feng**: Conceptualizing the entire paper, visualization, validation, review and editing, and funding acquisition. **Weihong Guo**: Review and editing, as well as funding acquisition. **Yutong Wu, Ting Zhu, and Kou Wu**: Literature search and data analysis, writing the original draft, drawing figures, and making tables. **Zean Wang, Sizhe Jiao, and Jiaxin Li**: Literature search and data analysis, investigation, formal analysis. All authors read and approved the final manuscript.

## CONFLICT OF INTEREST STATEMENT

The authors report no conflicts of interest in this work.

## Data Availability

Data sharing not applicable to this article as no datasets were generated or analysed during the current study.
